# Combining therapeutic hypothermia and primary coronary intervention in comatose survivors of ventricular fibrillation due to ST-elevation myocardial infarction

**DOI:** 10.1186/cc14240

**Published:** 2015-03-16

**Authors:** R Knafelj, M Noc

**Affiliations:** 1 Universty Clincal Center, Ljubljana, Slovenia

## Introduction

Primary percutaneous coronary intervention (PPCI) is the preferred reperfusion strategy for ST-elevation acute myocardial infarction (STEMI). In comatose survivors of cardiac arrest, mild induced hypothermia (MIH) improves neurological recovery.

## Methods

A total of 112 patients undergoing PPCI and MIH were compared with 32 comparable consecutive patients who underwent PPCI but no MIH. We hypothesized that combining both methods lead to better survival rate. MIH was induced (propofol, fentanyl, saline 4 ml/kg BW, 2°C) and maintained for 24 hours, targeting 32 to 34°C. Spontaneous rewarming was allowed (0.5°C).

## Results

There were no significant differences between the MIH and Control group in general characteristics, cardiac arrest circumstances and angiographic features. Except for decreases in heart rate during MIH, there was no difference between MIH and no MIH groups in arterial pressure, peak lactate (7.7 vs. 6.2 mmol/l; *P *= 0.36), need for vasopressors (57% vs. 41%; *P *= 0.09), aortic balloon counterpulsation (13% vs. 22%; *P *= 0.19), repeat cardioversion/defibrillation (17% vs. 25%; *P *= 0.30). There was lower incidence of inotropic use (36% vs. 59%; *P *= 0.01) and use of antiarrhythmics (11% vs. 53%; *P *= 0.002). There was no difference in FiO_2_ during mechanical ventilation and in renal function. See Table 1.

**Table 1:** Survival after 12 months.

	MIH	Control	*P *
CPC 1/2	50 (45%)	5 (15%)	0.002
CPC 3/4	0	0	NS
Cardio	20 (18%)	6 (19%)	0.95
CNS	18 (16%)	17 (53%)	0.00001

## Conclusion

Hospital survival with CPC 1/2 was significantly better in the MIH group (45% vs. 15%; *P *= 0.01). Our study clearly demonstrates that PPCI and MIH are feasible and may be combined safely in comatose survivors of ventricular fibrillation in STEMI setting. Such strategy improves survival with good neurological recovery.
